# Efficient Sustainable Tool for Monitoring Chemical Reactions and Structure Determination in Ionic Liquids by ESI-MS

**DOI:** 10.1002/open.201300022

**Published:** 2013-07-26

**Authors:** Levon L Khemchyan, Elena A Khokhlova, Marina M Seitkalieva, Valentine P Ananikov

**Affiliations:** [a] Zelinsky Institute of Organic Chemistry, Russian Academy of SciencesLeninsky Prospect 47, Moscow 119991 (Russia) E-mail: val@ioc.ac.ru

**Keywords:** carbohydrates, mass spectrometry, ionic liquids, MS/MS, peptides, reaction monitoring

## Abstract

An easy and convenient procedure is described for monitoring chemical reactions and characterization of compounds dissolved in ionic liquids using the well-known tandem mass spectrometry (MS/MS) technique. Generation of wastes was avoided by utilizing an easy procedure for analysis of ionic liquid systems without preliminary isolation and purification. The described procedure also decreased the risk of plausible contamination and damage of the ESI-MS hardware and increased sensitivity and accuracy of the measurements. ESI-MS detection in MS/MS mode was shown to be efficient in ionic liquids systems for structural and mechanistic studies, which are rather difficult otherwise. The developed ESI-MS/MS approach was applied to study samples corresponding to peptide systems in ionic liquids and to platform chemical directed biomass conversion in ionic liquids.

## Introduction

Electrospray ionization mass spectrometry (ESI-MS) is a well-known method widely used in polymer science,[1] carbohydrate[2] and peptide[3] analysis, protein structure determination,[4] catalysis,[5] and several other areas of practical applications.[6] From the discovery in the 1980s to today, ESI-MS disclosed plenty of opportunities for analysis of natural substances within molecular weight determinations, structural investigations and reaction mechanism studies. ESI is a “soft” ionization method that works with solutions at atmospheric pressure without or with negligible fragmentation of “parent” ions for many organic compounds. There are only few steps in sample preparation procedure, thus, making ESI-MS a quick analytical tool for obtaining qualitative and quantitative information about compounds and mixtures of interest.

Rapid development of modern chemistry in recent years has revealed the importance of new reaction media in addition to well-known organic solvents. One of the most fascinating findings concerns the outstanding scope of ionic liquids as reaction media, which showed tremendous growth in recent decades.[7–10] Due to their unique properties—virtually no vapor pressure, nonflammable, excellent thermal, mechanical and electrochemical stability, electrical conductivity, high polarity and exceptional dissolution properties—ionic liquids have become attractive and promising reaction media for different chemical transformations ranging from fine organic synthesis to processes of industrial importance.[7–10] Ionic liquids showed excellent results and recyclability for Heck reactions,[11] Friedel–Crafts-type transformations,[12] Michael additions,[13] hydroaminations of alkenes,[14] ring-closing metatheses[15] and many other important organic synthesis procedures.[16] Important to note, ionic liquids were proposed to be well-suitable “green” solvents for peptide synthesis[17] and biomass conversion.[10]

In spite of several promises and expectations concerning ionic liquids, recent studies have clearly shown that ionic liquids by no means are “intrinsically green”. Important issues to consider in this regard are toxicity of ionic liquids and environmental impact.[18] Ionic liquids can only become “green” if used appropriately, which takes into account the overall impact of the chemical process and environmental persistence. This challenge facilitates development of new, more sophisticated tools dealing with ionic liquid research.

The properties of ionic liquids that are advantageous for practical applications made it difficult to analyze these reaction mixtures by conventional analytical methods. High viscosity, conductive ionic character and absorption of radiofrequencies deteriorate the quality of high-resolution NMR spectra in ionic liquids.[19, 9] Recently, our group has reported a special NMR approach for the determination of molecular structures and monitoring of chemical reactions directly in ionic liquids.[20]

In the case of mass spectrometry the problem appears to be even more complicated. On the one hand, mass spectrometry with different ionization techniques (e.g., fast atom bombardment (FAB), electron ionization (EI), atmospheric-pressure chemical ionization (APCI), electrospray ionization (ESI)) provided a wealth of valuable information about ionic liquids itself, and their applications for example for the detection of reaction intermediates.[21, 22] For instance, ionic liquids were used as an effective “charged tag” for ESI-MS interception of key catalytic species involved in various chemical transformations.[22a]

**Scheme 1:**
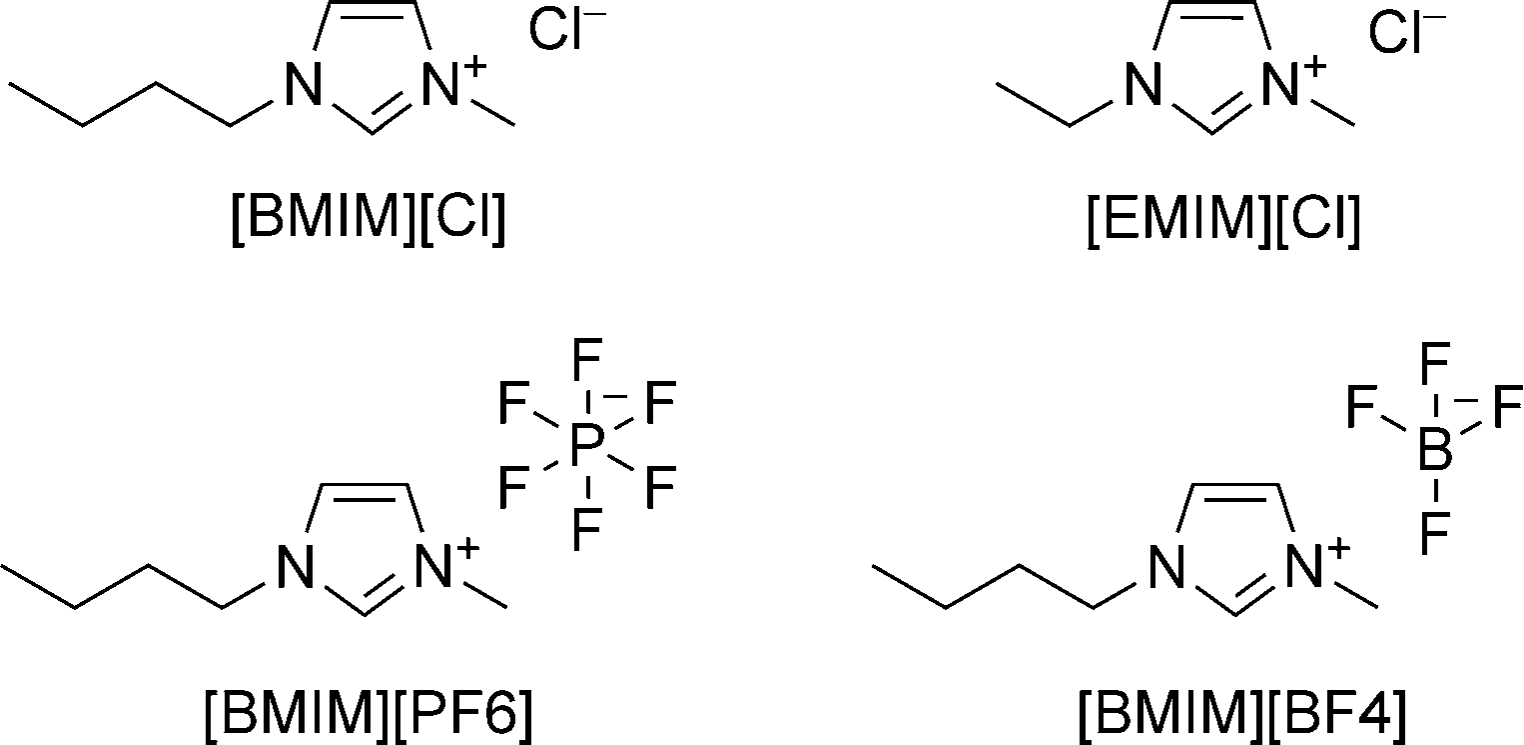
Structures of 1-butyl-3-methylimidazolium chloride ([BMIM][Cl]), 1-ethyl-3-methylimidazolium chloride ([EMIM][Cl]), 1-butyl-3-methylimidazolium hexafluorophosphate ([BMIM][PF_6_]) and 1-butyl-3-methylimidazolium tetrafluoroborate ([BMIM][BF_4_]).

On the other hand, a problem appears when ionic liquid serves only as solvent and is not bound to components of the reaction mixture. Indeed, since ionic liquid systems consist of ionized species at normal conditions under room temperature the ESI-MS spectra are dominated by intensive peaks of ionic liquid components in positive (cation) and negative (anion) ion modes (Scheme 1).[21, 22] In most cases the solute molecules dissolved in ionic liquids for carrying out the reaction can hardly be observed over the background of these highly ionized species, especially taking into account that the amount of solute is typically 10–100 times lower than the amount of solvent itself. Other plausible problems include a probable damage of MS hardware (ion transfer stage and other parts of the spectrometer) because of hardly removable contamination with ionic liquids and a decreasing detector lifetime (i.e., microchannel plate type) with extremely intensive ion beams generated from the ionic liquid.

Currently, analysis of reaction mixtures in ionic liquids requires careful purification before the analytical measurements in order to separate the compounds of interest from the ionic liquid. In spite of many promising green applications of technologies based on ionic liquids, current implementation and development of reactions in ionic liquids suffer from the limitations in analytical methods and separation procedures. Conventional isolation techniques (e.g., extraction, chromatography) are significantly time consuming and require organic solvents, later on released as wastes into the environment. What is really critical is that such preliminary treatment of ionic liquid systems is applicable only to stable compounds and might otherwise lead to chemical modifications or degradation of several types of species. Purification is unacceptable for mechanistic studies, since detection of unstable or reactive intermediates becomes impossible. Clearly, a useful practical approach should be developed to overcome these limitations and to perform direct ESI-MS analysis of ionic liquid systems. Excellent articles on the topic of mass spectrometric characterization of ionic liquids have appeared in recent years and demonstrated several fascinating opportunities in this field.[21, 22]

Herein, we describe a rapid and efficient procedure to monitor reactions in ionic liquids using ESI-MS by simple tuning of the MS instrument and avoiding damage of the hardware. The ESI-MS measurements are the analytical tool of choice to study ionic liquid systems due to a well-established structure resolving power and extremely high sensitivity.

## Results and Discussion

**Scheme 2:**
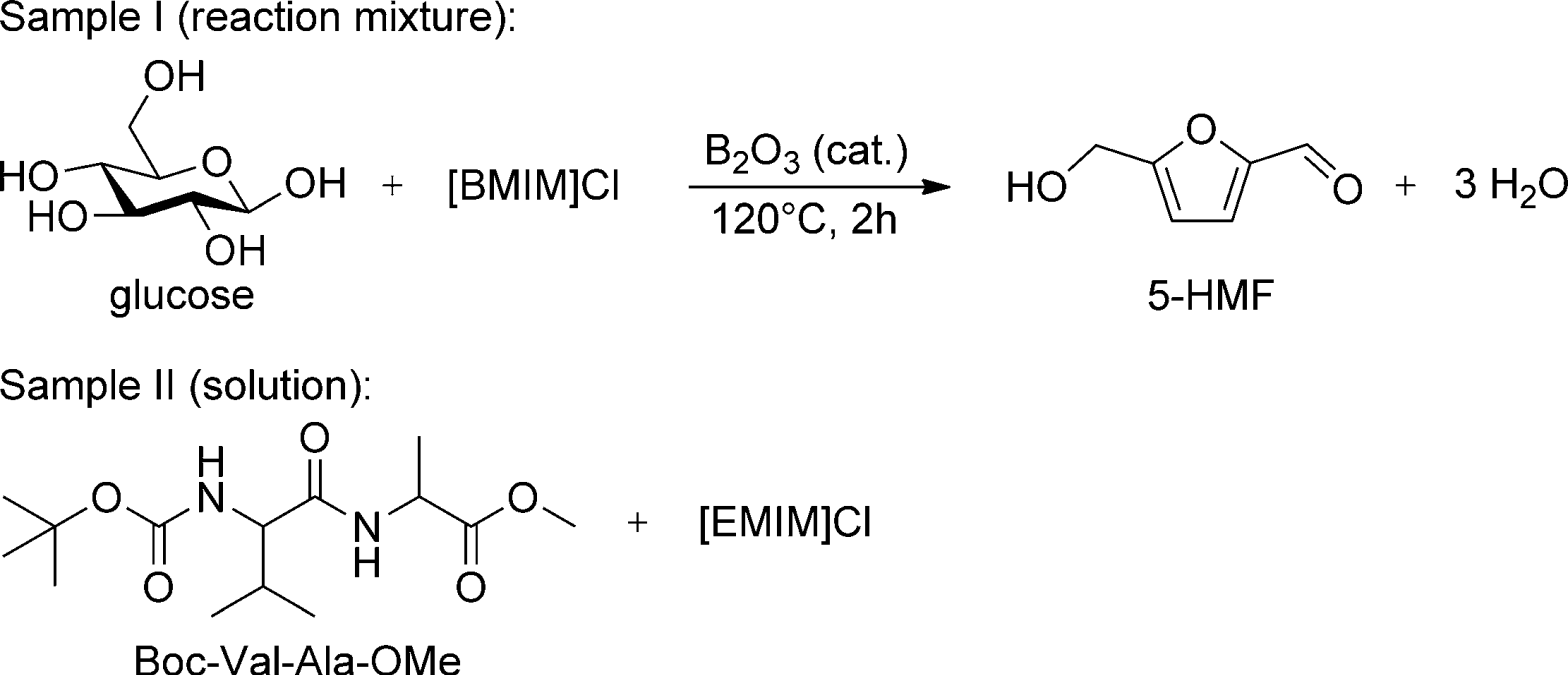
Studied carbohydrate conversion reaction (sample I) and peptide solution (sample II).

To evaluate the performance of ESI-MS measurements we have chosen two practically important systems in ionic liquids (Scheme 2). Sample I is a reaction mixture obtained after conversion of glucose to 5-(hydroxymethyl)furfural (5-HMF) carried out in 1-butyl-3-methylimidazolium chloride ([BMIM][Cl]). This sample corresponds to a real chemical system studied in the frameworks of developing of biomass conversion technologies.[10, 20, 23] Sample II is a solution of dipeptide Boc-Val-Ala-OMe in 1-ethyl-3-methylimidazolium chloride ([EMIM][Cl]), mimicking a typical system in peptide chemistry, which is a rapidly developing area of application of ionic liquids.[24]

An aliquot (0.6 mg) of each sample was completely dissolved in 1.5 mL of CH_3_CN and then diluted 10 000 times in the case of sample I and 100 000 times in the case of sample II with the same solvent. The concentrations of obtained solutions were in the order of 10^−14^–10^−15^ mol μL^−1^ for solutes and 10^−13^–10^−14^ mol μL^−1^ for ionic liquid (Table 1).

**Table 1:** Concentrations of ionic liquid’s cation and dissolved components of samples I and II

Sample	Species	Initial chemical system		ESI-MS sample
		Mass [g]	*c* in ionic liquid [mol μL^−1^]	*c* in CH_3_CN [mol μL^−1^]
	[BMIM]^+^	0.52	6.2×10^−6^	2.0×10^−13^
I	glucose	0.04	3.7×10^−7^	1.2×10^−14^
	5-HMF	0.03	4.0×10^−7^	1.3×10^−14^
	[EMIM]^+^	0.48	7.5×10^−6^	2.5×10^−14^
II	Boc-Val-Ala-OMe	0.05	2.9×10^−7^	1.0×10^−15^

**Figure 1:**
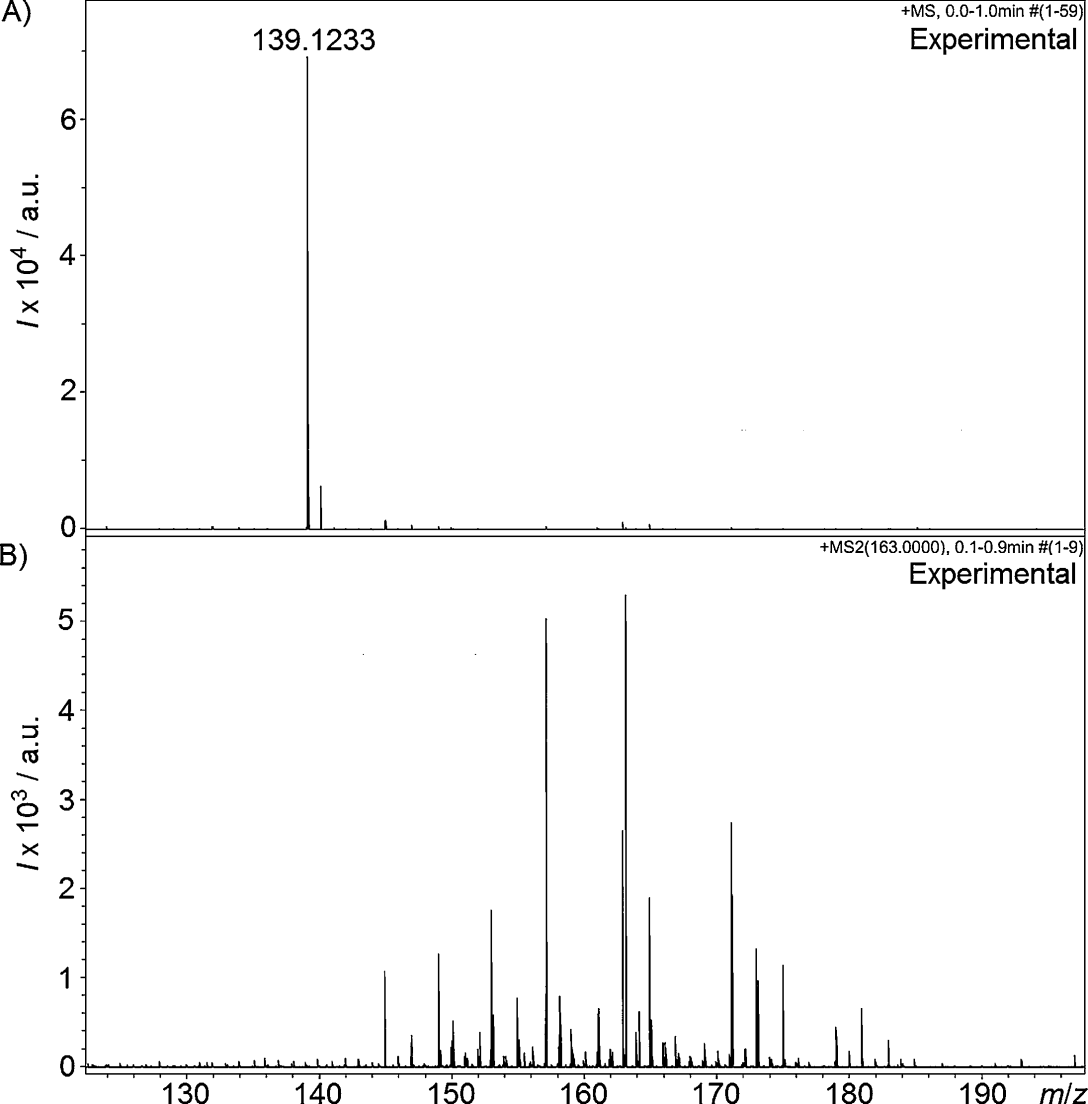
Representative spectra of the reaction mixture of sample I A) acquired in MS mode, B) acquired in MS/MS mode.

A representative mass spectrum of sample I registered in the MS mode is shown on Figure 1 A. Only an intensive (>60 000 units) signal of [BMIM]^+^ was observed in the ESI-MS spectrum at *m*/*z*=139.1233 with six to seven other negligible trace signals.[25] The signal of the product of interest, 5-HMF, was detected as a low-intensity trace impurity (<400 units; measured *m*/*z*=149.0228 for [C_6_H_6_O_3_+Na]^+^).[26] A rather large error of *Δ*=13.0 ppm was observed for the registered signal of 5-HMF (calculated *m*/*z*=149.0209 for [C_6_H_6_O_3_+Na]^+^), which is outside of the commonly acceptable error interval of <5 ppm. A more complicated problem was faced in the attempt to detect the unreacted part of the starting material of the studied reaction. The signal of glucose was indistinguishable from the noise with extremely low intensity with no more than 30–35 units.[26] As expected, regular ESI-MS spectra did not lead to characteristic measurements of the ionic liquid system: neither the starting material, nor the product of the reaction can be detected with reliable accuracy.

Repeated consecutive injections of the ionic liquid-containing samples at the MS mode may have a negative influence on the hardware, even with a concentration of the ionic liquid<0.5 pmol μL^−1^. Contamination of the instrument with ionic liquid species requires aborting the operation and performing system clean up. It should be pointed out that the spectrum in MS mode can be recorded only as a representative example. This experiment should not be repeated several times to avoid the risk of damage to the MS instrument detector and contamination of ion transfer stage and other parts of the hardware. Therefore, contamination and possible damage of the hardware are important limiting factors for carrying out ESI-MS measurements on ionic liquid systems, besides the low intensity and large error of the detected signals.

To avoid hardware contamination, we have carried out the measurements using tandem mass spectrometry (i.e., MS/MS mode).[27] The MS/MS mode, in contrast to MS mode, was designed to obtain and register fragments of parent ion(s) of interest. In a nutshell, this strategy works due to analytical quadrupole that can be adjusted to pass only a certain ion mass (parent ion) or a defined mass range (parent ions). In the collision cell the isolated ions can be fragmented through collision induced dissociation (CID) by collision with neutral gas molecules (i.e., nitrogen or argon). The degree of fragmentation is proportional to the value of collision energy and can be easily controlled. What is most important for the studied ionic liquid system: if collision energy is set to small values of 0–8 eV no (or negligible) fragmentation of parent ions will occur. This provides an outstanding opportunity to filter out the unwanted signal of the ionic liquid and to allow the parent ions of the studied compounds to reach the detector. It should be pointed out that MS/MS mode and CID are well-known techniques in mass-spectrometric detection,[27] however to the best of our knowledge they were not systematically applied to study ionic liquid systems.

We have successfully applied the MS/MS mode to detect the compounds of interest in the studied sample (5-HMF, glucose, ionic liquid) with complete removal of unwanted [BMIM]^+^ ions from the whole ion beam. The MS/MS mode with quadrupole adjusted to the range of *m*/*z*=142–184 allowed us to retrieve good quality spectra and prevented negative influence of [BMIM]^+^ contaminant on the detector and other parts of the MS instrument (Figure 1 B). A number of signals with good resolution and intensity have appeared within the studied interval, whereas no signal of [BMIM]^+^ (*m*/*z*=139.1233) was observed (cf. Figure 1 A and 1 B).

**Figure 2:**
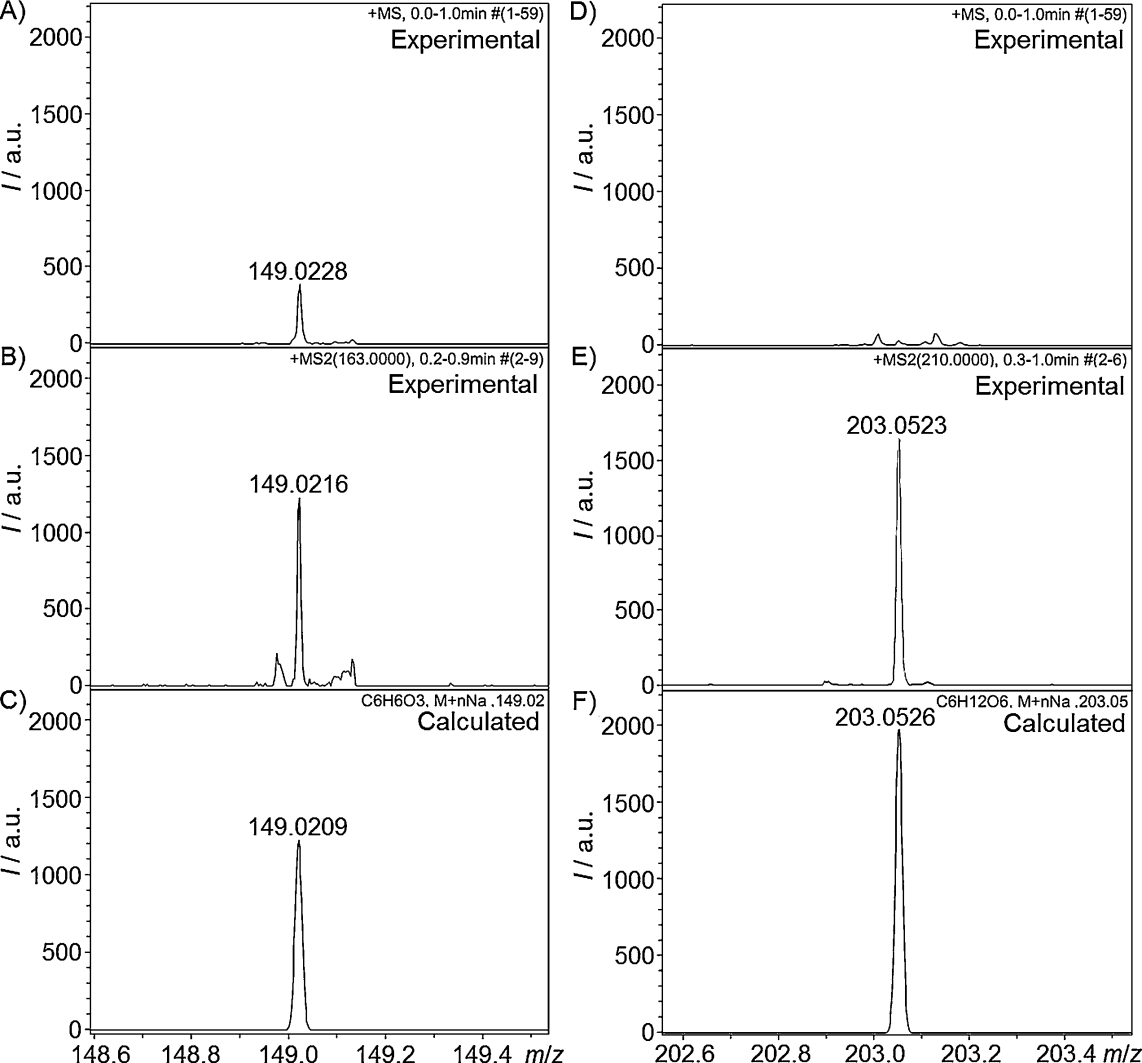
Enlarged sections of the mass spectra of sample I acquired in A, D) MS mode, B, F) MS/MS mode, and calculated spectra for C) [C_6_H_6_O_3_+Na]^+^ and F) [C_6_H_12_O_6_+Na]^+^.

In the MS/MS mode, the signal of 5-HMF product was detected with much higher intensity of 1250 units (Figure 2 B) compared to only 400 units in the MS mode (Figure 2 A). The error in the determination of the *m*/*z* value was reduced approximately three times to *Δ*=4.7 ppm, which is within the acceptable interval. The intensity of the 5-HMF signal was further increased to >2500 units by recording the spectrum in MS/MS mode with an adjusted mass range parameter, and the error in *m*/*z* determination in this case was decreased to *Δ*=2.0 ppm.[28]

The signal of glucose was not observable in MS mode (Figure 2 D), however it was easily detected in MS/MS mode (Figure 2 E). A good intensity of >1500 units was observed, and a small error of *Δ*=1.5 ppm in the determination of *m*/*z* value was found by comparison of experimental and calculated spectra (Figure 2 E and 2 F).

Thus, the measurements in MS/MS mode allowed to analyze reactions mixtures and to detect both reactant and product. A number of other signals were also observed in the ESI-MS spectra (possibly: impurities, intermediates or side products) indicating future possibilities of detailed characterization and mechanistic studies of this reaction.[29] Detailed analysis of the other signals is outside the scope of the present study, since here we focus on the development of a reliable analytical methodology.

To verify the scope of the developed analytical approach, we have also studied sample II, containing Boc-Val-Ala-OMe peptide dissolved in [EMIM][Cl]. Standard operation in MS mode yielded a spectrum with very low intensity signal of Boc-Val-Ala-OMe (180 units, *m*/*z*=325.1740 for [C_14_H_26_N_2_O_5_+Na]^+^). Analysis of the same sample in MS/MS mode led to significant increase in the signal intensity of up to 1400 units. The error in the determination of *m*/*z* value decreased from *Δ*=2.0 to 0.0 ppm. Representative ESI mass spectra of the Boc-Val-Ala-OMe peptide in the MS and MS/MS modes are shown in the Supporting Information (see Figure S4).

The scope of the developed approach was verified for samples I and II, as well as for the other peptides (samples III–V; Table 2). Comparison of the conventional measurements in MS mode and the developed approach using MS/MS mode has clearly shown superior performance of the latter for studying ionic liquid systems (Table 2). Indeed, in a conventional mode, it was not possible to distinguish the signals of some compounds due to spectral noise or low intensity signals. The MS/MS mode provided much better results for all samples in terms of intensity of signals and accuracy of the measurements. To the best of our knowledge, the feasibility of ESI-MS/MS detection of glucose, 5-HMF and peptides in ionic liquids was carried out for the first time in the concentration range of 0.001–0.01 pmol μL^−1^ after dilution.

**Table 2:** ESI-MS measurements for samples I–V in MS and MS/MS modes

Sample	Compd	Obsd ion	MS Mode		MS/MS Mode	
			*I* [a.u.]	*Δ* [ppm]	*I* [a.u.]	*Δ* [ppm]
	Glucose	[C_6_H_12_O_6_+Na]^+^	n.d.^[a]^	–	1600	1.5
I	5-HMF	[C_6_H_6_O_3_+Na]^+^	380	13.0	1250	4.7
II	Boc-Val-Ala-OMe	[C_14_H_26_N_2_O_5_+Na]^+^	180	2.0	1400	0.0
III	Boc-Ala-Val-OMe	[C_14_H_26_N_2_O_5_+Na]^+^	n.d.^[a]^	–	800	2
IV	Boc-Ala-Ala-Val-OMe	[C_17_H_31_N_3_O_6_+Na]^+^	300	1	1520	0.8
V	Boc-Val-Val-Ala-OMe	[C_19_H_35_N_3_O_6_+Na]^+^	150	11	2000	0.5

[a] n.d.=not detectable.

**Figure 3:**
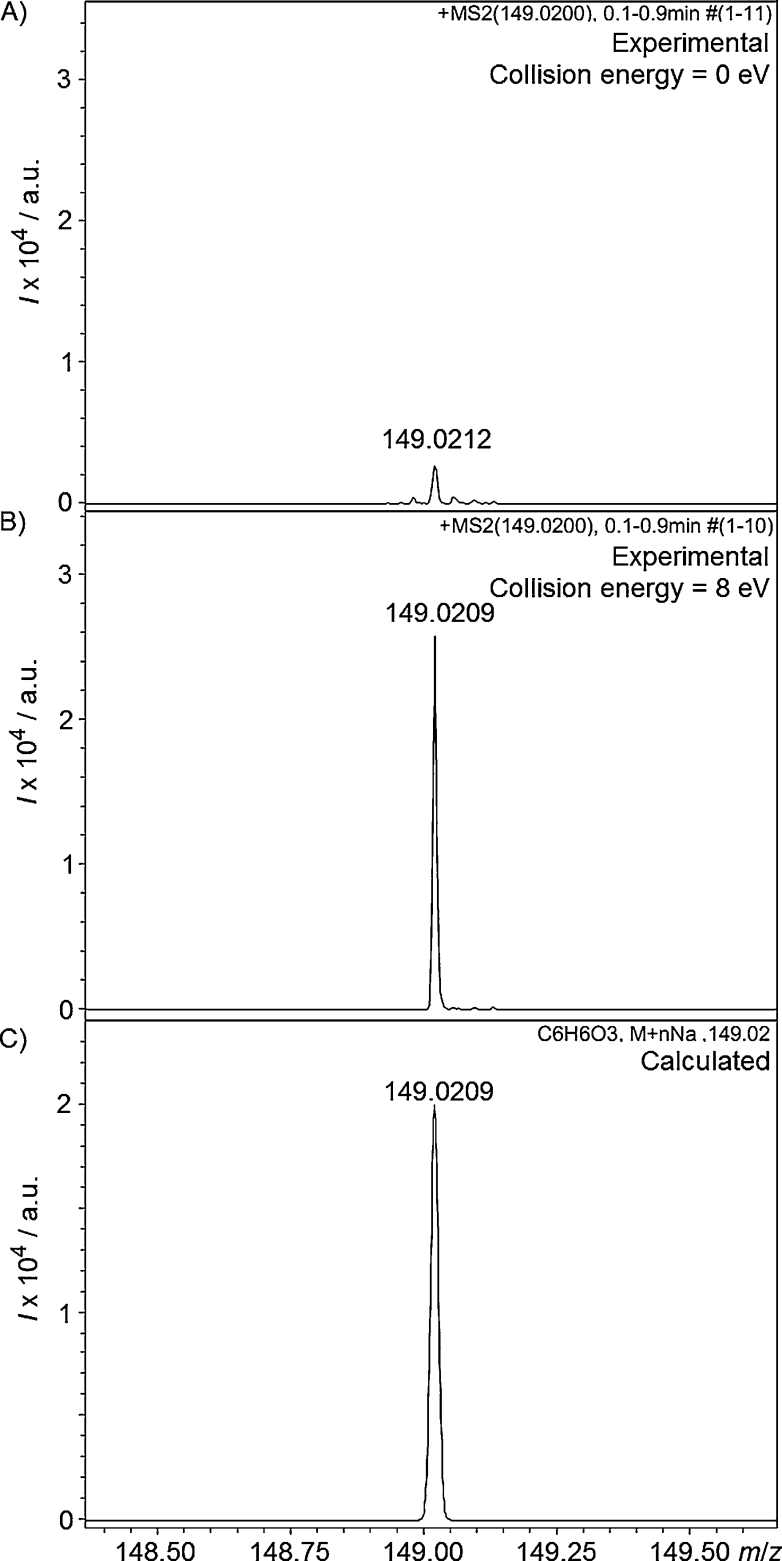
Enlarged sections of the mass spectra of sample I acquired in MS/MS mode (148.5–149.5 *m*/*z*) with collision energy set to A) 0 eV and B) 8 eV, and C) the calculated spectrum for [C_6_H_6_O_3_+Na]^+^.

We have demonstrated the principal applicability of ESI-MS measurements for ionic liquid samples. The final important point is to study the ability to tune MS/MS mode for better performance. A slight increase of collision energy from 0 to 8 eV led to noticeable gain in signal intensity, better accuracy of *m*/*z* determination and noise suppression. In fact, collision energy represents not only the degree of ion fragmentation, but also serves as the driving force for ion transfer.[30] The spectrum of sample I registered in MS/MS mode for detection of 5-HMF [C_6_H_6_O_3_+Na]^+^ has shown an excellent intensity (>25 000 units) upon applying collision energy of 8 eV (Figure 3). The error in *m*/*z* determination was decreased to *Δ*=0.0 ppm (Figure 3).

## Conclusions

**Figure 4:**
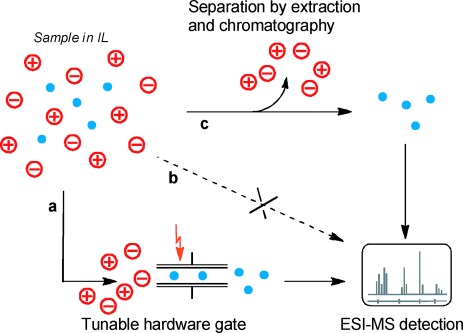
ESI-MS spectra of a) MS/MS mode described in the present study for filtering out of unwanted signals, b) direct analysis of the sample (not recommended because of probable spectrometer damage), c) conventional procedure that requires purification of the sample prior to the measurements.

In the present study, we describe a useful approach to carrying out tandem mass spectrometry (i.e., ESI-MS/MS) studies of ionic liquid solutions. The ionic liquid samples and reaction mixtures can be directly utilized for MS measurements using internal electronic gate available in all modern electrospray spectrometers (Figure 4 A, path a). Analytical quadrupole is used to filter out unwanted ions and separation of the species of interest. The filtration of the ions is carried out inside the mass spectrometer, the procedure is very fast (<1 millisecond) and does not require extra solvents. The described approach is superior to direct analysis (may cause hardware damage; Figure 4 B, path b) and to conventional separation (time-consuming and waste-generating stages; Figure 4 C, path c).

Thus, ESI-MS measurement in MS/MS mode is an attractive rapid method to monitor reactions in ionic liquid media, excluding the necessity of prior purification or extraction (no wastes and fast analysis) and minimizing the risk of a damage of the MS instrument hardware. Another valuable advantage is a noticeable increase of signal intensity. The method revealed a possibility of getting structural information under 0.01 pmol μL^−1^ concentration of the dissolved molecules. All the measurements described in the present study can be carried out on routinely available instruments and do not require any hardware modifications.

Taking into account the high sensitivity and structure resolving power of ESI-MS measurements, the developed MS approach can be of crucial importance for mechanistic studies as well as routine characterization of ionic liquid solutions and chemical transformations therein. We anticipate further studies on the application of ESI measurements in MS/MS mode in ionic liquid systems.

## Experimental Section

**General:** The detailed experimental method for conversion of glucose to 5-HMF using B_2_O_3_ as promoter (preparation of sample I) as described in previously[20] and a detailed procedure is given in the Supporting Information.

The experimental procedure for the synthesis and purification of dipeptide Boc-Val-Ala-OMe is described in the Supporting Information. Sample II was prepared by mechanical stirring the dipeptide (0.050 g) and ionic liquid (0.634 g) in a glass vial at 80 °C for 30 min. An aliquot (0.6 mg) of sample II was taken directly after heating before it was cooled to RT. A similar procedure was used for the other peptide samples.

CH_3_CN (HPLC grade) for ESI-MS experiments was purchased from Merck and used as supplied. All samples for ESI-MS experiments were prepared in 1.5 mL Eppendorf tubes. All plastic disposables used in sample preparation (Eppendorf tubes and tips) were washed with CH_3_CN before use.

**ESI-MS experiments:** High resolution mass spectra were measured on a Bruker maXis instrument using electrospray ionization (ESI) with MS and MS/MS modes. The measurements were performed in a positive ion mode (interface capillary voltage: 4500 V). “Tune low” method operating with a scan range from *m*/*z* 50 to *m*/*z* 300 was used for analysis of sample I; “tune wide” method operating with a scan range from *m*/*z* 250 to *m*/*z* 3000 was used for analysis of samples II–V. External calibration was performed with Electrospray Calibrant Solution (Fluka). A direct syringe injection was used for all analyzed solutions in CH_3_CN (flow rate: 3 μL min^−1^). All spectra were collected for 1 min. Nitrogen was applied as a dry and collision gas; interface temperature was set at 180 °C. The spectra were processed by using Bruker Data Analysis 4.0 software package.

**ESI-MS/MS operation settings:** The parameters for operation in MS/MS mode for sample I (Figures 1 B and 2 B) are described below as a representative example. The settings for the MS/MS mode of all samples used in the present study are given in the Supporting Information.

Isolated mass parameter corresponding to the center of the desired *m*/*z* interval was set to 163; isolated width corresponding to the width of the interval was set to 42. The isolated width was adjustable from 0.1 to 300 *m*/*z* depending on the *m*/*z* region of interest. Acceptable values of collision energy were in the range of 0–8 eV. In source collision induced dissociation (ISCID energy) refers to MS3 mode, the ISCID energy should be set to 0 eV. Acquisition factor was set to 6.7 to define the number of time of flight events. In a typical case, the acquisition factor within the interval from 5 to 10 is enough to get good quality spectrum, although it may be set to a larger value at lower concentration of detecting compounds in the analyzed probe. Note, some care should be taken during MS/MS spectral data interpretation, since MS/MS experimenst may also result in appearance of electronic noise, which, however, can be distinguished from real signals.[31]

## Supplementary material

As a service to our authors and readers, this journal provides supporting information supplied by the authors. Such materials are peer reviewed and may be re-organized for online delivery, but are not copy-edited or typeset. Technical support issues arising from supporting information (other than missing files) should be addressed to the authors.

## References

[1a] Hakkarainen M (2012). Mass Spectrometry of Polymers – New Techniques.

[1b] Hanton SD (2001). Chem. Rev.

[1c] Montaudo MS (2002). Mass Spectrom. Rev.

[2a] Zaia J (2004). Mass Spectrom. Rev.

[2b] Ruhaak LR, Deelder AM, Wuhrer M (2009). Anal. Bioanal. Chem.

[2c] Zaia J (2009). Mass Spectrom. Rev.

[2d] Matamoros Fernández LE (2007). Carbohydr. Polym.

[3a] Biniossek ML, Schilling O (2012). Proteomics.

[3b] Zhu PH, Bowden P, Zhang D, Marshall JG (2011). Mass Spectrom. Rev.

[3c] Banerjee S, Mazumdar SJ (2010). J. Mass Spectrom.

[3d] Pisano E, Cabras T, Montaldo C, Piras V, Inzitari R, Olmi C, Castagnola M, Messana I (2005). Eur. J. Oral Sci.

[4a] Bruneaux M, Rousselot M, Leize E, Lallier FH, Zal F (2008). Curr. Protein Pept. Sci.

[4b] Kebarle P, Verkerk UH (2009). Mass Spectrom. Rev.

[4c] Konermann L, Stocks BB, Pan Y, Tong X (2010). Mass Spectrom. Rev.

[4d] Aebersold R, Goodlett DR (2001). Chem. Rev.

[4e] Davis MT, Lee TD (1998). J. Am. Soc. Mass Spectrom.

[5a] Cole RB (1997). Electrospray Ionization Mass Spectrometry: Fundamentals, Instrumentation and Applications.

[5b] Henderson W, McIndoe JS (2005). Mass Spectrometry of Inorganic, Coordination and Organometallic Compounds.

[5c] Santos LS (2010). Reactive Intermediates: MS Investigations in Solution.

[5d] Dell′Anna MM, Mastrorilli P, Nobile CF, Calmuschi-Cula B, Englert U, Peruzzini M (2008). Dalton Trans.

[5e] Belyakov PA, Kadentsev VI, Chizhov AO, Kolotyrkina NG, Shashkov AS, Ananikov VP (2010). Mendeleev Commun.

[6a] Marshall AG, Hendrickson CL (2008). Ann. Rev. Anal. Chem.

[6b] Cottingham K (2005). Anal. Chem.

[6c] Gross JH (2004). Mass Spectrometry: A Textbook.

[6d] Bluck L, Volmer DA (2008). Spectroscopy.

[7a] Welton T (1999). Chem. Rev.

[7b] Weingärtner H (2008). Angew. Chem.

[b01] (2008). Angew. Chem. Int. Ed.

[7c] Hallett JP, Welton T (2011). Chem. Rev.

[7d] Haumann M, Riisager A (2008). Chem. Rev.

[7e] Scholten JD, Leal BC, Dupont J (2012). ACS Catal.

[8a] Plechkova NV, Seddon KR (2008). Chem. Soc. Rev.

[8b] Earle MJ, Plechkova NV, Seddon KR (2009). Pure Appl. Chem.

[8c] Ferguson JL, Holbrey JD, Ng S, Plechkova NV, Seddon KR, Tomaszowska AA, Wassell DF (2012). Pure Appl. Chem.

[9a] Wasserscheid P, Keim W (2000). Angew. Chem.

[b02] (2000). Angew. Chem. Int. Ed.

[9b] Ananikov VP (2011). Chem. Rev.

[9c] Pensado AS, Pádua AAH (2011). Angew. Chem.

[b03] (2011). Angew. Chem. Int. Ed.

[9d] Olivier-Bourbigou H, Magna L, Morvan D (2010). Appl. Catal. A.

[10a] Zakrzewska ME, Bogel-Łukasik E, Bogel-Łukasik R (2011). Chem. Rev.

[10b] Rosatella AA, Simeonov SP, Frade RFM, Afonso CAM (2011). Green Chem.

[10c] Ståhlberg T, Fu W, Woodley JM, Riisager A (2011). ChemSusChem.

[10d] Lima S, Antunes MM, Pillinger M, Valente AA (2011). ChemCatChem.

[10e] Gallezot P (2012). Chem. Soc. Rev.

[10f] Wang H, Gurau G, Rogers RD (2012). Chem. Soc. Rev.

[11] Handy ST, Okello M, Dickenson G (2003). Org. Lett.

[12] Snelders DJM, Dyson PJ (2011). Org. Lett.

[13] Sarkar D, Bhattarai R, Headley AD, Ni B (2011). Synthesis.

[14] Yin P, Loh T-P (2009). Org. Lett.

[15] Wakamatsu H, Saito Y, Masubuchi M, Fujita R (2008). Synlett.

[16a] Aggarwal VK, Emme I, Mereu A (2002). Chem. Commun.

[16b] Kume Y, Qiao K, Tomida D, Yokoyama C (2008). Catal. Commun.

[16c] Conte V, Elakkari E, Floris B, Mirruzzo V, Tagliatesta P (2005). Chem. Commun.

[16d] Virtanen P, Salmi T, Mikkola J-P (2009). Ind. Eng. Chem. Res.

[16e] Pinto AC, Moreira Lapis AA, da Silva BV, Bastos RS, Dupont J, Neto BAD (2008). Tetrahedron Lett.

[16f] Rosa JN, Afonso CAM, Santos AG (2001). Tetrahedron.

[16g] Angelini G, De Maria P, Chiappe C, Fontana A, Gasbarri C, Siani G (2009). J. Org. Chem.

[17a] Ternois J, Ferron L, Coquerel G, Guillen F, Plaquevent J-C

[17b] Vallette H, Ferron L, Coquerel G, Guillen F, Plaquevent J-C (2006). Arkivoc.

[17c] Vallette H, Ferron L, Coquerel G, Gaumont A-C, Plaquevent J-C (2004). Tetrahedron Lett.

[18a] Welton T (2011). Green Chem.

[18b] Stasiewicz M, Mulkiewicz E, Tomczak-Wandzel R, Kumirska J, Siedlecka EM, Gołebiowski M, Gajdus J, Czerwicka M, Stepnowski P (2008). Ecotoxicol. Environ. Saf.

[18c] Matzke M, Stolte S, Thiele K, Juffernholz T, Arning J, Ranke J, Welz-Biermann U, Jastorff B (2007). Green Chem.

[18d] Peric B, Marti E, Sierra J, Cruañas R, Iglesias M, Garau MA (2011). Environ. Toxicol. Chem.

[18e] Ventura SPM, de Barros RLF, Sintra T, Soares CMF, Lima ÁS, Coutinho JAP (2012). Ecotoxicol. Environ. Saf.

[18f] Ventura SPM, Marques CS, Rosatella AA, Afonso CAM, Gonçalves F, Coutinho JAP (2012). Ecotoxicol. Environ. Saf.

[19a] Carper WR

[19b] Giernoth R, Koel M (2009). Ionic Liquids in Chemical Analysis.

[20] Khokhlova EA, Kachala VV, Ananikov VP (2012). ChemSusChem.

[21a] Dyson PJ, Henderson MA, McIndoe JS, Plechkova NV, Rogers RD, Seddon KR (2010). Ionic Liquids: From Knowledge to Application.

[21b] Dyson PJ, McIndoe JS, Zhao D (2003). Chem. Commun.

[22a] Dupont J, Eberlin MN (2013). Curr. Org. Chem.

[22b] Neto BAD, Meurer EC, Galaverna R, Bythell BJ, Dupont J, Cooks RG, Eberlin MN (2012). J. Phys. Chem. Lett.

[22c] Law WS, Chen H, Ding J, Yang S, Zhu L, Gamez G, Chingin K, Ren Y, Zenobi R (2009). Angew. Chem.

[b04] (2009). Angew. Chem. Int. Ed.

[23] Vennestrøm PNR, Osmundsen CM, Christensen CH, Taarning E (2011). Angew. Chem.

[b05] (2011). Angew. Chem. Int. Ed.

[24a] Plaquevent J-C, Levillain J, Guillen F, Malhiac C, Gaumont A-C (2008). Chem. Rev.

[24b] Buchfink R, Tischer A, Patil G, Rudolph R, Lange C (2010). J. Biotechnol.

[24c] Byrne N, Wang L-M, Belieres J-P, Angell CA (2007). Chem. Commun.

[24d] Huang JL, Noss ME, Schmidt KM, Murray L, Bunagan MR (2011). Chem. Commun.

[24e] Constatinescu D, Herrmann C, Weingärtner H (2010). Phys. Chem. Chem. Phys.

[24f] Guillen F, Brégeon D, Plaquevent J-C (2006). Tetrahedron Lett.

[24g] Moniruzzaman M, Nakashima K, Kamiya N, Goto M (2010). Biochem. Eng. J.

[24h] Miloslavina AA, Leipold E, Kijas M, Stark A, Heinemann SH, Imhof D (2009). J. Pept. Sci.

[27a] Hunter AP, Games DE (1994). Rapid Commun. Mass Spectrom.

[27b] Santa T (2011). Biomed. Chromatogr.

[27c] Kushnir MM, Rockwood AL, Bergquist J (2010). Mass Spectrom. Rev.

[27d] Sabino AA, Machado AHL, Correia CRD, Eberlin MN (2004). Angew. Chem.

[b06] (2004). Angew. Chem. Int. Ed.

[27e] Niessen WMA (2011). Mass Spectrom. Rev.

[30] Mayer PM, Poon C (2009). Mass Spectrom. Rev.

[31] Mujezinovic N, Raidl G, Hutchins JRA, Peters J-M, Mechtler K, Eisenhaber F (2006). Proteomics.

