# Supplementary material for: Efficient Sustainable Tool for Monitoring Chemical Reactions and Structure Determination in Ionic Liquids by ESI-MS
Source: ChemistryOpen. 2013 Jul 26;2(5-6):208–14. doi: 10.1002/open.201300022 (PMC3892193; doi:10.1002/open.201300022)
Supplement: Supplementary file 1 [file open0002-0208-SD1.pdf]

ChemistryOPEN

Supporting Information

© 2013 The Authors. Published by Wiley-VCH Verlag GmbH & Co. KGaA, Weinheim

Efficient Sustainable Tool for Monitoring Chemical Reactions and Structure Determination in Ionic Liquids by ESI-MS

Levon L. Khemchyan, Elena A. Khokhlova, Marina M. Seitkalieva, and Valentine P. Ananikov*[a]

open_201300022_sm_miscellaneous_information.pdf

CONTENT

Experimental procedure for conversion of glucose to 5-HMF 2
Experimental procedure for synthesis of N-tert-butoxycarbonyl-valinylalanine methyl ester (Boc-Val-Ala-OMe) 2
ESI-MS2 operation settings for Samples I -V 2
Figure S1. A) The enlarged part of MS spectrum of the the reaction mixture of Sample I acquired in MS mode and B) the calculated spectra for $[C_6H_6O_3 + Na]^+$ 3
Figure S2. A) The enlarged part of MS spectrum of the the reaction mixture of Sample I acquired in MS mode and B) the calculated spectra for $[C_6H_{12}O_6 + Na]^+$. 3
Figure S3. A) The enlarged part of MS spectrum of the the reaction mixture of Sample I acquired in MS2 mode (a) and B) the calculated spectra for $[C_6H_6O_3 + Na]^+$. MS2 mode in the 148.5-149.5 m/z range. “Isolated Mass” set to 149.0; “Isolated Width” set to 1; “Collision Energy” set to 0 eV; “Acquisition Factor” set to 5.5 4
Figure S4. A) The enlarged parts of MS spectra of the Sample II acquired in MS mode. B)in MS2 mode) and C) The calculated spectrum for $[C_{14}H_{26}N_2O_5 + Na]^+$. MS2 mode in the 324-326 m/z range. “Isolated Mass” set to 325; “Isolated Width” set to 2; “Collision Energy” set to 0 eV; “Acquisition Factor” set to 10 5
Figure S5. ^{1}H NMR spectra ($CDCl_3$, 600 MHz) of N-Boc-Val-Ala-OMe. 6
Figure S6 . ^{13}C NMR spectra ($CDCl_3$, 600 MHz) of N-Boc-Val-Ala-OMe. 6

Experimental procedure for conversion of glucose to 5-HMF

In a typical experiment, a reaction mixture containing glucose (0.56 mmol, 0.10 g), B_2O_3 (0.28 mmol, 19.5 mg) and [BMIM]Cl (3.73 mmol, 0.652 g) was transferred under Ar into a glass tube. The resulting sample was placed in an oil bath at 120ºC. The reaction was carried out at 120ºC with continuous stirring (800 rpm); see ref.[19] for more details.

Experimental procedure for synthesis of N-tert-butoxycarbonyl-valinylalanine methyl ester (Boc-Val-Ala-OMe)

To a stirred and ice-cooled solution of N-tert-butoxycarbonyl-L-valine (0.217 g, 1 mmol) in dichlormethane (3 ml) dicyclohexylcarbodiimide (0.206 g, 1 mmol) was added. After 20 min the mixture was combined with solution of the alanine methyl ester hydrochloride (0.154 g, 1.1 mmol) in dry dichlormethane (3 ml), which was previously neutralized with triethylamine (0.14 ml, 1 mmol). Finally 4-dimethylaminopyridine (0.012 g, 0.1 mmol) was added to the mixture. The mixture was stirred at 0° C for 1 h, then overnight at room temperature. The reaction mixture was filtered, concentrated in vacuo, suspended in cold ethyl acetate and filtered. The filtrate was washed successively with 1 N hydrochloric acid (3×5 ml), brine (2×5 ml), 1 M potassium bicarbonate (3×5 ml) and brine (2×5 ml). Then the solution was dried over sodium sulfate, filtered and concentrated under reduced pressure. N-tert-butoxycarbonyl-valinylalanine methyl ester was isolated by flash chromatography (7:3 hexanes/ethyl acetate) to yield the dipeptide as a white solid (0.263 g, 87 %).

ESI-MS2 operation settings for Samples I -V

Sample I, Figure 2e: MS2 mode in the 202-218 m/z range. “Isolated Mass” set to 210; “Isolated Width” set to 16; “Collision Energy” set to 0 eV; “Acquisition Factor” set to 10.

Sample I, Figure 3a: MS2 mode in the 148.5-149.5 m/z range. “Isolated Mass” set to 149.02; “Isolated Width” set to 1; “Collision Energy” set to 0 eV; “Acquisition Factor” set to 5.5.

Sample I, Figure 3b: MS2 mode in the 148.5-149.5 m/z range. “Isolated Mass” set to 149.02; “Isolated Width” set to 1; “Collision Energy” set to 8 eV; “Acquisition Factor” set to 5.5.

Sample II, Table 2: MS2 mode in the 324-326 m/z range. “Isolated Mass” set to 325; “Isolated Width” set to 2; “Collision Energy” set to 0 eV; “Acquisition Factor” set to 10 (Fig. S4).

Sample III, Table 2: MS2 mode in the 324-326 m/z range. “Isolated Mass” set to 325; “Isolated Width” set to 2; “Collision Energy” set to 8 eV; “Acquisition Factor” set to 10.

Sample IV, Table 2: MS2 mode in the 395-397 m/z range. “Isolated Mass” set to 396; “Isolated Width” set to 2; “Collision Energy” set to 8 eV; “Acquisition Factor” set to 8.

Sample V, Table 2: MS2 mode in the 423-425 m/z range. “Isolated Mass” set to 424; “Isolated Width” set to 2; “Collision Energy” set to 8 eV; “Acquisition Factor” set to 8.

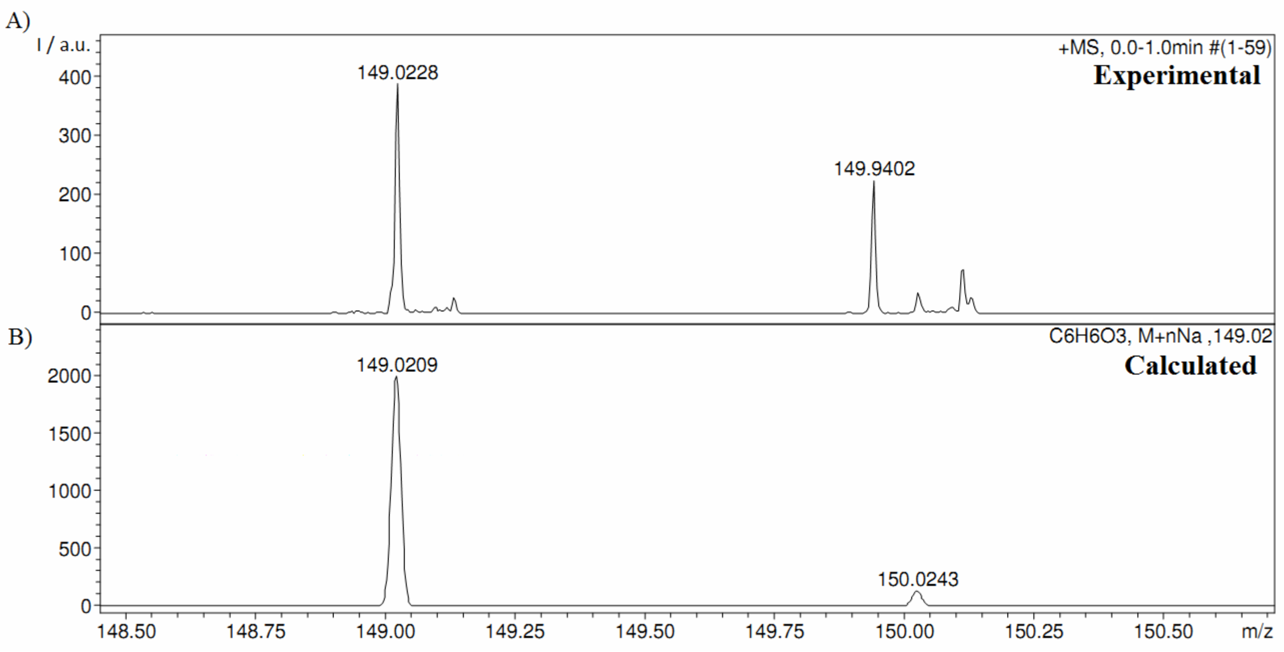

Figure S1. A) The enlarged part of MS spectrum of the the reaction mixture of Sample I acquired in MS mode and B) the calculated spectra for $[C_6H_6O_3 + Na]^+$.

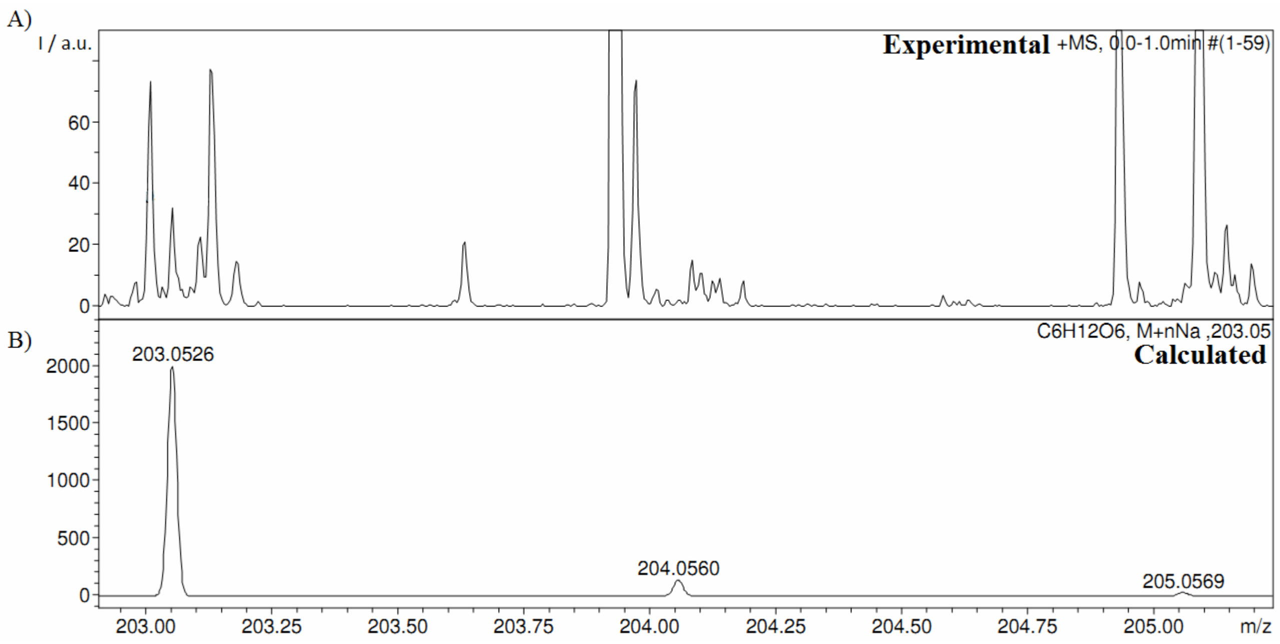

Figure S2. A) The enlarged part of MS spectrum of the the reaction mixture of Sample I acquired in MS mode and B) the calculated spectra for $[C_6H_{12}O_6 + Na]^+$.

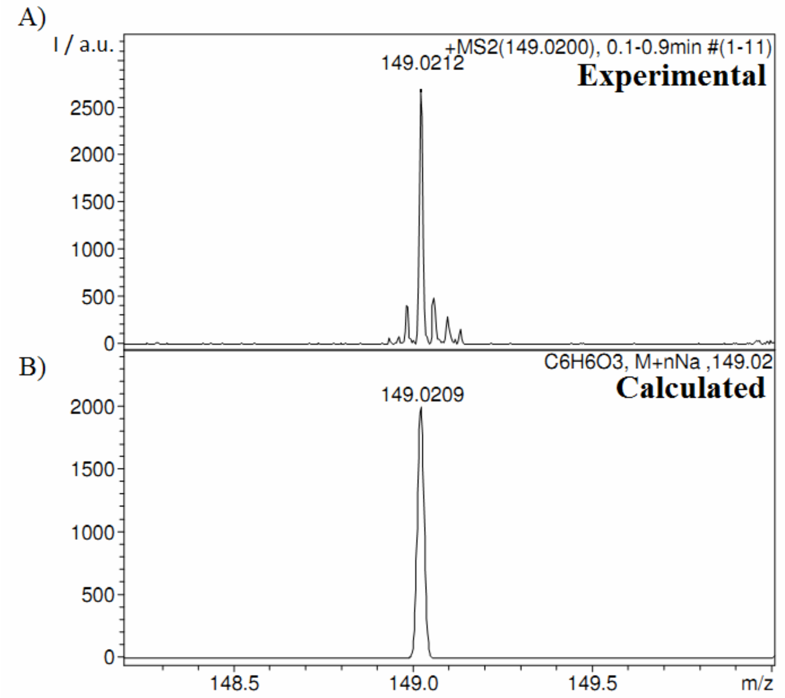

Figure S3. A) The enlarged part of MS spectrum of the the reaction mixture of Sample I acquired in MS2 mode (a) and B) the calculated spectra for $[C_6H_6O_3 + Na]^+$. MS2 mode in the 148.5-149.5 m/z range. “Isolated Mass” set to 149.0; “Isolated Width” set to 1; “Collision Energy” set to 0 eV; “Acquisition Factor” set to 5.5.

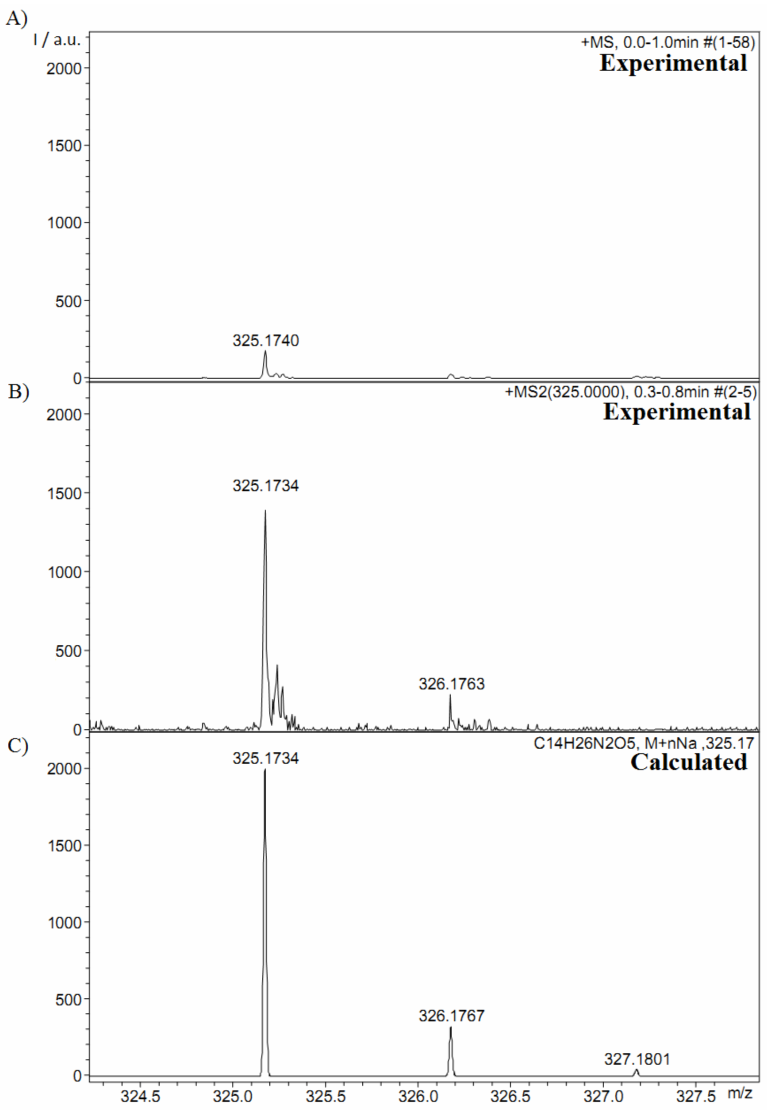

Figure S4. A) The enlarged parts of MS spectra of the Sample II acquired in MS mode. B)in MS2 mode) and C) The calculated spectrum for $[C_{14}H_{26}N_2O_5 + Na]^+$. MS2 mode in the 324-326 m/z range. “Isolated Mass” set to 325; “Isolated Width” set to 2; “Collision Energy” set to 0 eV; “Acquisition Factor” set to 10.

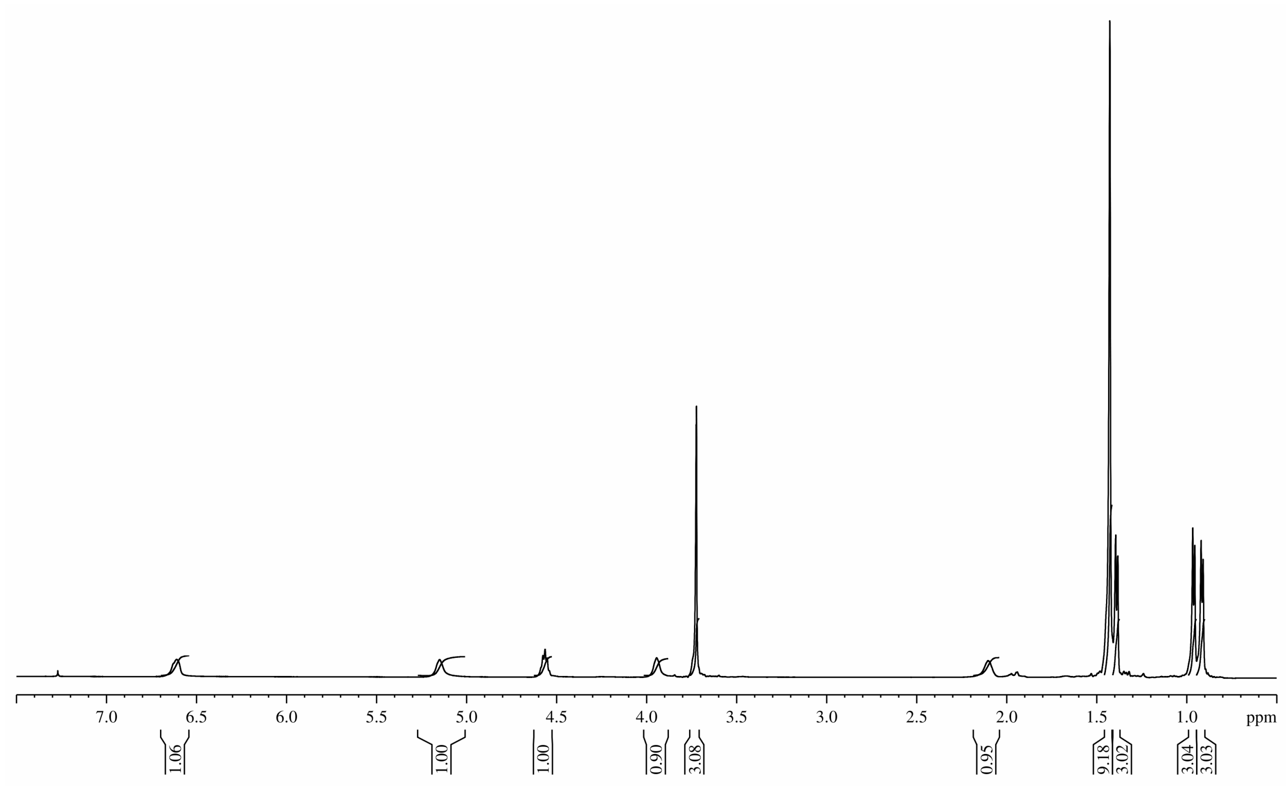

Figure S5. ^{1}H NMR spectra ($CDCl_3$, 600 MHz) of N-Boc-Val-Ala-OMe.

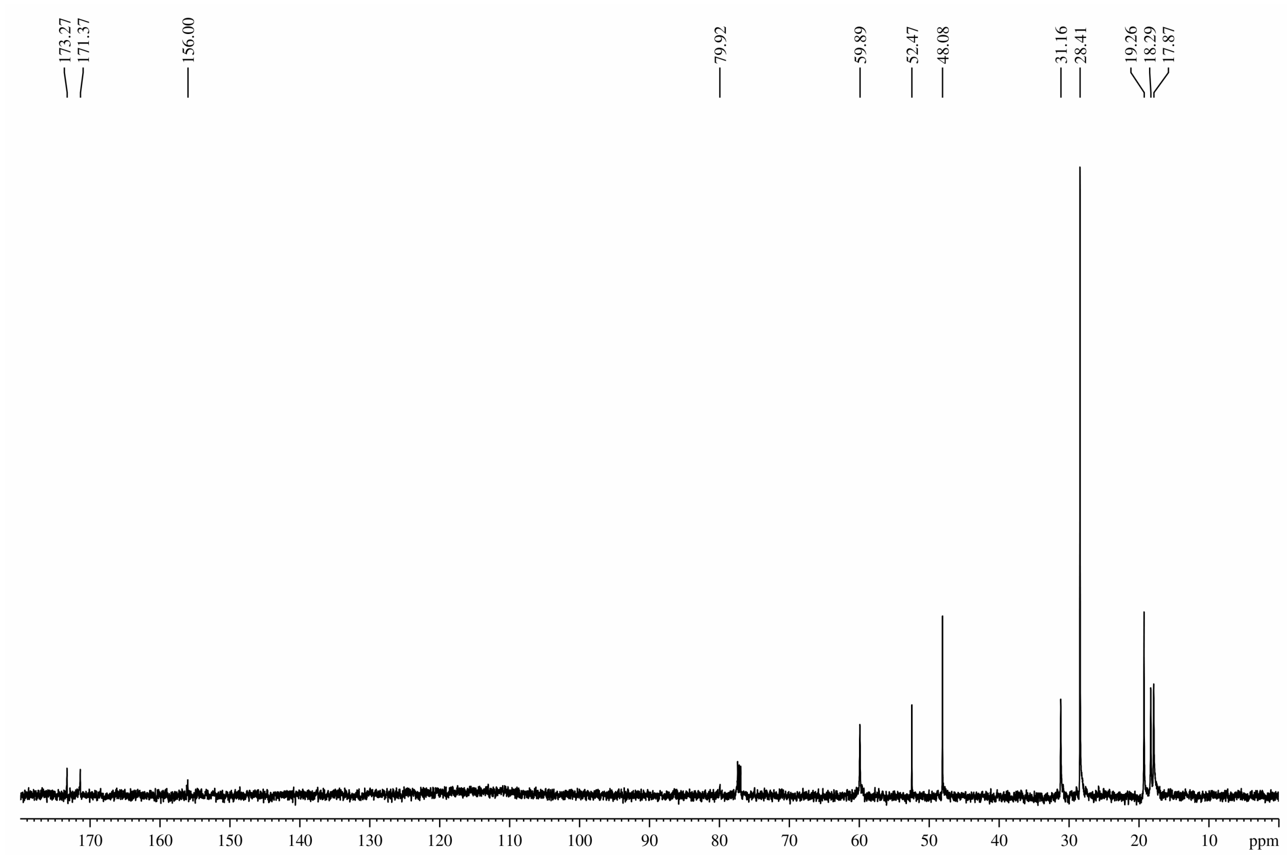

Figure S6 . ^{13}C NMR spectra ($CDCl_3$, 600 MHz) of N-Boc-Val-Ala-OMe.
